# Centering peers in design and training for a peer-delivered contingency management program for self-identified harm reduction and treatment goals

**DOI:** 10.1186/s12954-025-01213-z

**Published:** 2025-05-06

**Authors:** Linda Peng, Erin Stack, Alexis Cooke, Bryan Hartzler, Ryan Cook, Gillian Leichtling, Christi Hildebran, Judith Leahy, Kelsey Smith Payne, Lynn Kunkel, Kim Hoffman, P. Todd Korthuis

**Affiliations:** 1 Department of Medicine, Section of Addiction Medicine, Oregon Health & Science University, 3181 SW Sam Jackson Park Road, Mail Code UHN30, Portland, OR 97239 USA; 2 Comagine Health, Portland, OR USA; 3 Addictions, Drug and Alcohol Institute, University of Washington, Seattle, WA USA; 4 Health Systems Division, Oregon Health Authority, Portland, OR USA; 5 Oregon Health & Science University-Portland State University School of Public Health, Portland, OR USA

**Keywords:** Behavior therapy, Reinforcement, Stimulants, Substance-related disorders, Psychosocial intervention, Community-based participatory research, Motivation, Methamphetamine

## Abstract

**Background:**

Novel strategies are needed to engage people who use stimulants into the continuum of addiction care. Contingency management (CM) is the most effective intervention for stimulant use disorder and may engage non-treatment-seeking populations, especially when delivered by peer recovery support specialists (peers). We describe development and training for a novel peer-delivered CM program for stimulant use harm reduction and treatment engagement.

**Methods:**

We used a community based participatory research (CBPR) process to develop a CM program focused on self-identified goals for harm reduction and treatment engagement. A steering committee of peers guided study design, CM rewards, schedule, and incentivized goals. Peers completed coaching-to-criterion of six CM skills based on the CM Competence Scale (CMCS), then completed a one-on-one roleplay with a standardized patient. Coaches rated peer performance of each CMCS skill according to its Likert scale (1 = Very Poor to 7 = Excellent) and an a priori rating criterion of 4 (‘adequate’). Roleplays included feedback and a ‘replay’ of skills, if necessary.

**Results:**

The steering committee devised two CM interventions: an enhanced standard-of-care incentivizing peer visits ($20 for weekly peer visits) and an intervention that additionally incentivized self-directed goals ($20 for weekly peer visits and $30 for completed goal-related activities). Self-identified goal-related activities were chosen through a collaborative process and organized into 6 domains: (1) overdose/overamping prevention (2) substance use supports/treatment (3) daily living/housing (4) education/employment (5) mental/physical/spiritual health (6) social relationships. Forty-seven peers across nine peer-led organizations (three rural and six urban organizations across Oregon) completed CM training. All 47 peers met the a priori criterion in their roleplay, with seventeen (36%) requiring a ‘replay’ of a skill. Mean CMSC summary scores were 28.51 (SD 4.73) on the first attempt and 29.62 (SD 4.01) on the second attempt.

**Conclusions:**

PEER-CM (Peers Expanding Engagement in Stimulant Harm Reduction with Contingency Management) is among the first trials to use peer-delivered CM for stimulant use, incentivizing peer engagement and self-identified goals for harm reduction and treatment engagement. A CBPR approach strengthened the study design by incorporating peer guidance. Peers in this large, multisite sample demonstrated adequate CM delivery skills with acceptable fidelity following training.

*Trial Registration*

This study is registered at ClinicalTrials.gov (NCT 05700994). Registered 26 January, 2023.

**Supplementary Information:**

The online version contains supplementary material available at 10.1186/s12954-025-01213-z.

## Background

The drug overdose epidemic continues to devastate communities in the United States. Overdose deaths have risen exponentially related to fentanyl and the COVID-19 pandemic, with 2023 being the deadliest year ever for people who use drugs (PWUD) [1–4]. In Oregon, opioid overdose deaths increased from 280 deaths in 2019 to 1049 deaths in the first three quarters of 2023 [5]. Stimulant-involved overdose deaths are also rising rapidly, disproportionately affecting racial and ethnic minorities including Black/African American and American Indian/Alaskan Native populations [6, 7]. Co-use of stimulants (especially methamphetamine) and opioids is common and associated with greater overdose risk compared to opioid or stimulant use alone [8, 9]. To address this epidemic, more strategies are needed to engage people who use stimulants in harm reduction and treatment services.

Behavioral treatment is the gold standard for treating stimulant use disorder since there are no FDA-approved medications for stimulant use disorder, and medications have limited benefit [10–12]. Contingency management (CM) is an evidence-based intervention that utilizes a reward-based system as positive reinforcement and is effective for all substance use disorders (SUD) [13]. CM is the most effective intervention for reducing stimulant use and also increases treatment retention and engagement [14]. Yet, evidence for CM is almost exclusively in treatment seeking populations and typically incentivizes abstinence from substance use, not reaching the majority of PWUD who are not seeking treatment [15–17]. Notably, the empirical basis for CM as an efficacious therapy for increasing treatment adherence rests on research trials in which CM was delivered by research personnel or traditional addiction treatment professionals. This leaves unanswered questions about potential roles for other professionals in CM delivery, who can contribute beneficially across the SUD care continuum.

One such group of professionals that increasingly populate the behavioral health workforce are peer recovery support specialists (peers), who (among many things) can uniquely engage PWUD who are not treatment seeking. Peers use their lived experience in addiction and recovery to gain trust and form personal connections with PWUD, providing encouragement and hope. Peers also focus on holistic goals, including harm reduction, that may not always fit into traditional SUD treatment settings which are typically more abstinence and recovery focused [18]. Through these connections, peer involvement improves substance use recovery outcomes and positively impacts lives of PWUD [19]. Peer-delivered CM has the potential to engage difficult to reach populations of PWUD, but is understudied [20]. Recent reports from the U.S. Department of Health and Human Services (HHS) and Substance Abuse and Mental Health Services Administration (SAMHSA) explicitly recommend against peers delivering CM, despite no evidence that peers cannot deliver CM effectively and in a manner consistent with the peer role of supporting self-identified goals [21, 22].

Peers and peer-led organizations provide essential harm reduction services that serve as a bridge to SUD treatment, but peers and PWUD are not always included in research design or the development of interventions intended to support PWUD [23]. Meaningful peer involvement in research design has the potential to more effectively address community needs and improve outcomes most important to PWUD [24]. Furthermore, very few studies have examined whether peers can be effectively trained to deliver CM. To address this gap in understanding of peer workforce capability, we used a community-based participatory research (CBPR) approach centering the needs of PWUD in designing a harm reduction and treatment engagement intervention. CBPR approaches actively engage communities affected by the research questions to inform study design [25, 26]. By involving peers with lived experience in addiction, we developed a CM intervention that is specifically tailored to meet the needs of PWUD.

The Peers Expanding Engagement in Stimulant Harm Reduction with Contingency Management (PEER-CM) study aims to test the effectiveness of peer-delivered CM for achieving harm reduction and treatment engagement goals to reduce overdose in people who use stimulants, targeting individuals who are non-treatment seeking [27]. This manuscript reports the CBPR process and training fidelity outcomes used to support peers in learning evidence-based CM skills for incentivizing client-directed harm reduction and treatment engagement goals.

## Methods

### Study design, setting, and participants

PEER-CM study design and protocol have been reported in detail, elsewhere [27]. This manuscript focuses on the CBPR process and training outcomes. Briefly, Oregon’s extensive network of community-based peer support service programs were offered to participate. Participating peer organizations were part of Oregon’s network of peer services that offer outreach, harm reduction, and treatment engagement to PWUD in 24 of Oregon’s 36 counties [28]. In Oregon, peers with lived experience in recovery from addiction are certified by the state after completion of an approved peer training curriculum [18]. Nine geographically diverse organizations were selected who manage 14 distinct peer “sites”. All sites were trained to implement a “standard-of-care” CM intervention that incentivized client engagement with peer visits. Sites were then trained and randomized to initiate client incentives for meeting self-identified harm reduction/treatment engagement goals in addition to peer visit incentives, with two sites implementing this approach every two months. Clients are eligible for enrollment if they are age 18 or older, used any stimulant in the past 30 days, and are willing to accept peer services. Clients are followed for six months to assess for self-reported overdose (primary outcome), meeting self-identified harm reduction goals, and engagement in treatment services (secondary outcomes). This study was approved by the Oregon Health & Science Institutional Review Board (IRB #24672).

### Community-based participatory research approach

The PEER-CM study was designed using a CBPR approach informed by principles of CBPR including: (1) facilitating collaborative partnerships between community and research partners, (2) tailoring the study design to meet the needs of the community and PWUD, and (3) promoting an empowering process for peers and peer supervisors to participate in development, implementation, evaluation, and dissemination [24, 25]. All participating peer organizations were invited to send one or two representatives to participate in the PEER-CM steering committee. Steering committee members across peer organizations included organizational leaders, peer supervisors, and peers. Meetings typically had 1–2 representatives from at least 6 peer organizations in attendance. The steering committee also included technical assistance and training partners and research partners from all partnering organizations.

The steering committee convened virtually for one-hour sessions to accommodate busy schedules and vast geographic spread across the state. The steering committee convened twice during the grant application development phase, every other week during the project planning phase (January 2023 to August 2023), and monthly during the implementation phase which began February 2024. The goal of the steering committee was to facilitate collaboration, support, and shared project leadership across implementation, technical assistance and training, and research staff to effectively design a CM intervention that supports people who use stimulants in achieving harm reduction goals and reducing opioid overdose and stimulant overamping. Steering committee members helped create study materials and provided feedback on PEER-CM study design and core elements of the PEER-CM program. PEER-CM program design was also informed by existing CM literature and expertise, ensuring adherence to core principles and concepts of CM (e.g. targeted behaviors are meaningful and objectively verifiable, rewards are provided consistently and accurately) [29, 30].

Steering committee members provided feedback on all data collection tools and project materials and helped answer the following study questions related to CM, identifying and documenting goals, and collecting data:

- What types of gift cards to provide?
- What should the incentive structure look like (i.e., number of gift cards, amount of gift cards, and frequency of gift cards)?
- What will the goal action planning process look like between peers and clients?
- What goals will be incentivized?
- What will overdose/overamping prevention discussions look like?
- What are the eligibility criteria for participating in PEER-CM?
- What will the PEER-CM fidelity review process look like?
- What will the data quality check process look like?

We used Basecamp, a cloud-based project management and collaboration tool, to share meeting materials and solicit asynchronous feedback from steering committee members. We kept a running list of upcoming questions in an Excel file on Basecamp, which included questions, date to discuss in meeting, facilitator to lead discussion, decision status (i.e., in preparation, posted, decided), and final decision when applicable. Meeting agendas with decision topics and any relevant documents to review or reference materials were posted one week prior to each meeting. Prior to each discussion, PEER-CM study staff outlined the decision-making process, including stating that we wanted to hear from all steering committee members and may call on people for their input directly. Steering committee members could provide feedback verbally, via chat, or via Zoom reactions (e.g., thumbs up, heart). PEER-CM study staff with expertise in CBPR approaches facilitated open discussions for each topic, summarized a final decision, and led a decision-making voting process, if needed. Decision-making voting was done using numbers presented in the chat from 1 to 8. Votes from 1 to 6 represented approval from enthusiastic support to meager support. Votes from 7 to 8 represented strong objection and would lead to continued discussion during current or future meetings. Meeting minutes with decisions were posted to Basecamp after each meeting. When feedback was related to document and material development, PEER-CM study staff walked through documents, collected feedback, integrated feedback between meetings, and then brought final documents to future meetings for steering committee approval.

### Contingency management training

Peers underwent a contingency management coaching-to-criterion training process where peers are “coached” or trained to an *a priori* fidelity benchmark for specific “criterion” or clinical skills. This CM coaching-to-criterion process has been proven to effectively train addiction clinicians, with higher CM skillfulness demonstrated to improve client outcomes [31, 32]. CM training included an overview of important CM concepts and coaching-to-criterion of specific CM skills based on six core domains of the validated Contingency Management Competence Scale (CMCS): (1) Informing the client of rewards, (2) Outlining the potential for future rewards, (3) Providing earned rewards, (4) Assessing client interest in rewards, (5) Providing praise and social reinforcement, and (6) Linking reward to goals [33]. These six core domains of the CMCS have high reliability, internal consistency, and durability [32, 34]. A two-hour CM skills group training session included demonstrations by trainers, practice roleplays by peers, and performance-based feedback from trainers and peers. Group training sessions were primarily in-person, with a virtual option for peers who could not attend the in-person trainings. Most peers (65%) attended an in-person training.

Following group training, each peer completed a virtual 20-min, one-on-one roleplay with a standardized patient in which peers were asked to demonstrate the six core domains of the CMCS in a simulated encounter. A CM trainer (LP, ES, AC, LK) observed each roleplay and provided feedback. Peers were given the opportunity to ‘replay’ specific skills if not adequately demonstrated on the first attempt. Trainers rated the six core domains of the CMCS in real time according to its Likert scale (1 = Very Poor to 7 = Excellent) and an *a priori* rating criterion of 4 (‘adequate’) for each skill domain [35]. All CM trainers underwent a train-the-trainer process, including practice and feedback on CMCS ratings, to ensure reliability and validity of CMCS ratings.

## Results

### PEER-CM study design development

Nine peer-led organizations (three rural and six urban organizations across Oregon) representing 14 sites and four partnering technical assistance and research organizations formed the steering committee. During the grant development stage, the peer-led organizations drove the decision to compare two peer-led CM approaches: (1) incentivizing peer visits as the enhanced standard-of-care and (2) incentivizing goal-related activities in addition to peer visits as the intervention. Peer-led organizations felt it was important that both approaches included CM rewards so that all participants could potentially be offered CM rewards regardless of study arm.

During the project planning phase, the steering committee finalized eligibility criteria, decided reward specifics (i.e., gift card types, amount, schedule), determined specific incentivized goal-related activities, provided input, and finalized the opioid overdose and stimulant overamping prevention materials (i.e., peer- and participant-facing prevention planning documents and wallet cards). During the implementation phase, the steering committee discussed study progress on enrollment targets and dissemination opportunities, described site-level challenges and successes, shared resources across sites, and continued to provide input on updated project materials and processes.

Importantly, steering committee peers highlighted the need to expand eligibility criteria from methamphetamine only to all stimulants, specifically to include crack and powder cocaine and prescription stimulant misuse. For CM rewards, peer-led organizations highlighted the importance of having gift cards to large grocery stores with a variety of products (e.g. Fred Meyer) that were accessible in the community. Peers also recommended incentivizing weekly peer engagement with $20 gift cards and having a higher reward amount of $30 for completed goal-related activities, feeling that this schedule would be the most motivating for participants (Table 1).

**Table 1:** PEER-CM incentives

**Standard-of-care** $20 for weekly peer visits (15 visits up to $300)
**Intervention** $20 for weekly peer visits (15 visits up to $300) $30 for completing goal-related activities (10 goals-related activities up to $300)

### Incentivized client-identified goals

Peers felt that incentivizing clients’ self-identified goals was an important way to support clients, reduce overdose, and increase engagement in treatment services. Peers identified numerous goals that are typically important to PWUD and emphasized the importance of having many options across different domains since clients’ goals can vary. Goals were organized into six domains: (1) Overdose/Overamping Prevention and Risk Reduction Planning, (2) Substance Use/Recovery Supports, (3) Daily Living, Routines, and Housing, (4) Education, Employment, and Finances, (5) Mental, Physical, and Spiritual Health, and (6) Social, Recreational, Relationships, and Family [27]. Guided by CM principles of rewarding specific objective behaviors and acknowledging that some goals require a multitude of steps, peer organizations decided to shift incentivized self-identified goals to rewarding self-identified goal-related activities that include steps taken (e.g., scheduling a SUD treatment appointment) toward larger goals (e.g., engaging in SUD treatment). The final list of goals and incentivized goal-related activities can be found in our protocol manuscript [27].

### Harm reduction materials

The steering committee identified the need for discussion guides around prevention of opioid overdose and stimulant overamping. Based on steering committee feedback, discussion guides, participant handouts, and fold-out wallet cards were developed that include educational information and self-selection of harm reduction actions that can be documented on the wallet card as a harm reduction plan. The materials used a discussion approach based on the REBOOT 2.0 overdose counseling intervention, which aimed to engage participants in a discussion about their opioid overdose experiences, strategies to reduce opioid overdose risk, and participant-specific risk reduction planning [36]. We adapted materials for use by peers and expanded to include stimulant overamping content and translated participant-facing materials to Spanish. Additional adaptations included development of a detailed script for peers and project-specific handouts and wallet cards for participants. Additional files show the fold-out wallet cards *[see 1_Wallet Cards]*, overdose prevention discussion guide *[see 2_Opioid Overdose Prevention Discussion Guide]*, overamping prevention discussion guide *[see 3_Stimulant Overamp Prevention Discussion Guide]*, opioid overdose handout *[see 4_Opioid Overdose Handout]*, and stimulant overamping handout *[see 5_Stimulant Overamp Handout]*.

### CM coaching-to-criterion training scores

As of May 2024, 47 peers and peer supervisors (who may provide back-up support if a peer is unavailable) from nine peer-led organizations completed the contingency management coaching-to-criterion training, including the one-on-one roleplay with a standardized patient. Thirty (64%) participants adequately demonstrated all six CM skills on the first attempt, receiving an *a priori* rating criterion of 4 (‘adequate’) or more on all six CMCS skill domains. Seventeen (36%) participants required a ‘replay’ of one or more skills. The two skills that most frequently required a ‘replay’ were providing earned rewards (n = 7, 15%) and assessing client interest in rewards (n = 7, 15%). The mean CMCS summary score was 28.51 (SD = 4.73, range = 21–40) on the first attempt and 29.62 (SD = 4.01, range = 24–40) on the second attempt. Mean final score and standard deviation (SD) for each CMCS domain were: (1) Informing the client of rewards (M = 4.89, SD = 0.67), (2) Outlining the potential for future rewards (M = 5.00, SD = 0.78), (3) Providing earned rewards (M = 4.85, SD = 1.06), (4) Assessing client interest in rewards (M = 5.09, SD = 1.00), (5) Providing praise and social reinforcement (M = 4.89, SD = 0.94), and (6) Linking reward to goals (M = 4.89, SD = 0.84).

### Peer workforce challenges

Workforce shortages in addiction and mental health are a significant barrier to increasing access to addiction treatment [37]. Many participating peer-led organizations experienced peer turnover and workforce shortages which affected peer training and implementation. As of May 2024, 30 peers and 25 peer supervisors or other organization administrative staff were involved in the PEER-CM project. Eight peers, five peer supervisors, and five other organization administrative staff completed all PEER-CM trainings and are no longer involved in the PEER-CM project. In addition, many other peers started but did not complete the PEER-CM training process. Of those no longer involved in PEER-CM for whom we have turnover information, some (n = 6) shifted to different internal positions and the remaining (n = 22) left the organizations completely. Reasons provided for leaving included difficulty of peer work on personal recovery, no longer being interested in peer work, moving to a different organization, and retiring. To ensure that all new peers are adequately trained in CM skills, peers completed CM training by watching previously recorded CM group trainings, then underwent one-on-one roleplays with a standardized patient (scores included above). Peers could also request additional in-person training for extra skills practice.

## Discussion

Although earlier-referenced HHS and SAMHSA reports cautioned against peer-delivered CM for lack of supporting evidence [21, 22], that cautionary recommendation acknowledged that ongoing research may influence future recommendations. Using a fidelity criterion employed in multiple implementation research trials with samples of traditional addiction professionals [32, 34, 38], our large, multisite sample of peers demonstrated capability to skillfully deliver CM. In addition to demonstrating competence in CM skills, peers felt that CM skills were natural to incorporate into client encounters and were excited about being able to offer additional positive rewards. CM skills pair well with peer work as peers often elicit client goals and use positive rewards for relationship building (e.g. meeting for coffee) [18]. Since peers are uniquely positioned to engage non-treatment seeking PWUD in harm reduction and treatment engagement, peer-delivered CM has the potential to increase motivation and improve engagement for people outside of treatment settings. Our data from this ongoing trial make clear that peers can competently deliver CM with fidelity, and we look forward to future reporting opportunities wherein the clinical effectiveness of these CM interventions may be addressed.

Very few studies have used CM to incentivize harm reduction strategies [39, 40] even though CM—particularly when delivered by peers—has the potential to improve engagement in the face of rising stimulant use. Peer-led organizations informed design of this client-centered CM program though a CBPR process. Involving PWUD and peers in harm reduction and treatment engagement initiatives ensures that interventions are designed to center the needs of PWUD [23, 24, 41]. The input of the steering committee was instrumental in shaping the PEER-CM intervention, as committee members provided guidance on reward magnitudes and delivery schedules to maximize client motivation and engagement. Steering committee contributions to incentivized goals also helped ensure a comprehensive, meaningful goal list, building trust with peers responsible for implementation and enhancing participant engagement. The composition and role of the steering committee evolved over different phases of the project from proposal development to implementation. Open and transparent discussions facilitated these transitions, ensuring continued alignment with study goals. In some instances, ad hoc one-on-one site meetings with organizations helped clarify the purpose and value of their engagement in the steering committee meetings. Interestingly, peer recommendations, such as the importance of rewarding peer engagement and individualized client-identified goals, largely align with CM expert guidelines for adapting CM for stimulant use harm reduction [42]. The PEER-CM study will examine whether this novel approach of using CM for harm reduction increases treatment engagement and reduces overdose.

In the U.S., access to CM is improving with recent changes allowing CM funding from SAMHSA and some states allowing CM through Medicaid Section 1115 waivers [22, 43]. However, CM for stimulant use disorder remains underutilized across most of the U.S. Key barriers include financial constraints, philosophical opposition, and concerns about time and staffing needed to deliver CM effectively [30]. Peer-delivered CM is a promising strategy to mitigate these challenges by broadening the scope of services provided by peers within the SUD treatment continuum, helping address concerns related to staff availability and workload. However, the peer workforce faces challenges, including high turnover rates and workforce shortages, which mirror long-standing trends in the broader mental health and addiction workforce [37, 44]. Strengthening the addiction workforce will require targeted strategies, including expanding access to evidence-based interventions such as CM and equipping a wide range of addiction professionals with the skills to deliver these treatments effectively.

Study findings should be interpreted considering a few limitations. First, Oregon’s robust peer support network may limit generalizability of these findings to states with less developed peer support infrastructure [28]. The implementation of PEER-CM also coincided with Oregon’s decriminalization and subsequent recriminalization of drug possession, which likely influences how peers address client goals within the context of CM [45]. Future qualitative studies will explore these dynamics in greater depth. Second, while our CBPR process included multiple peer-led organizations across Oregon, it did not include people actively using drugs, as all peers were in recovery for at least 2 years in compliance with Oregon peer recovery support specialist requirements. Although peers with lived experience have expertise in the needs of PWUD, there may be additional harm reduction needs or goals that our intervention design did not include. We are tracking intervention feedback and will be exploring these gaps in qualitative interviews with peers and clients. Third, skills-focused performances with a standardized patient do not guarantee that standard is met in every encounter with actual patients in real-world practice. That said, standardized patient methodology does provide opportunity to tailor an assessment to skills of interest, and to provide a consistent stimulus (e.g. a fair test) in that assessment across a group of trainees. To encourage fidelity to CM skills over the course of the study, peers receive refresher trainings on CM skills at least once a year and additional re-trainings on request. The PEER-CM study will evaluate the effectiveness of this CM intervention in a real-world setting. Finally, peer turnover and workforce shortages also made ensuring consistent and adequate training a challenge and may affect fidelity to the PEER-CM intervention. We consequently offer recurrent trainings on a regular basis.

## Conclusions

Using a CBPR approach, the PEER-CM study was designed to use peer-delivered CM to engage non-treatment seeking people who use stimulants in harm reduction and treatment engagement goals—providing rewards for peer engagement and completing self-identified goal-related activities. The CBPR process played a pivotal role in informing and strengthening the study design by incorporating peer guidance to ensure that the PEER-CM intervention effectively addresses the needs of PWUD. PEER-CM will explore how incentivizing self-identified goals supports harm reduction and treatment engagement. After CM training on evidence-based CM skills, a large, multisite sample of peers demonstrated adequate CM delivery with acceptable fidelity. Peer-delivered CM has the potential to incentivize non-treatment seeking people to engage in harm reduction and treatment services that reduce opioid overdoses and stimulant overamping.

## Additional material

1_Wallet Cards.pptx—Wallet cards: Fold out educational wallet cards for opioid overdose prevention and stimulant overamping prevention.

2_Opioid Overdose Prevention Discussion Guide.docx—Opioid overdose discussion guide: Opioid overdose prevention discussion guide for peers.

3_Stimulant Overamp Prevention Discussion Guide.docx—Stimulant overamping discussion guide: Stimulant overamping prevention discussion guide for peers.

4_Opioid Overdose Handout.docx—Opioid overdose handout: Opioid overdose prevention handout for participants.

5_Stimulant Overamp Handout.docx—Stimulant overamping handout: Stimulant overamping prevention handout for participants.

## Supplementary Information

Additional file 1.

Additional file 2.

Additional file 3.

Additional file 4.

Additional file 5.

## Abbreviations

PWUD: People who use drugs
CM: Contingency management
SUD: Substance use disorder
Peers: Peer recovery support specialists who have lived experience in addiction
CBPR: Community based participatory research
PEER-CM: Peers expanding engagement in stimulant harm reduction with contingency management
CMCS: Contingency Management Competence Scale

## Declarations

### Availability of data and materials

The data supporting the conclusions of this article are available upon request to Linda Peng, pengli@ohsu.edu.
