# Supplementary material for: Centering peers in design and training for a peer-delivered contingency management program for self-identified harm reduction and treatment goals
Source: Harm Reduct J. 2025 May 6;22(Suppl 1):72. doi: 10.1186/s12954-025-01213-z (PMC12057027; doi:10.1186/s12954-025-01213-z)
Supplement: Supplementary file 1 — Additional file 1. [file 12954_2025_1213_MOESM1_ESM.pptx]

## Slide 1
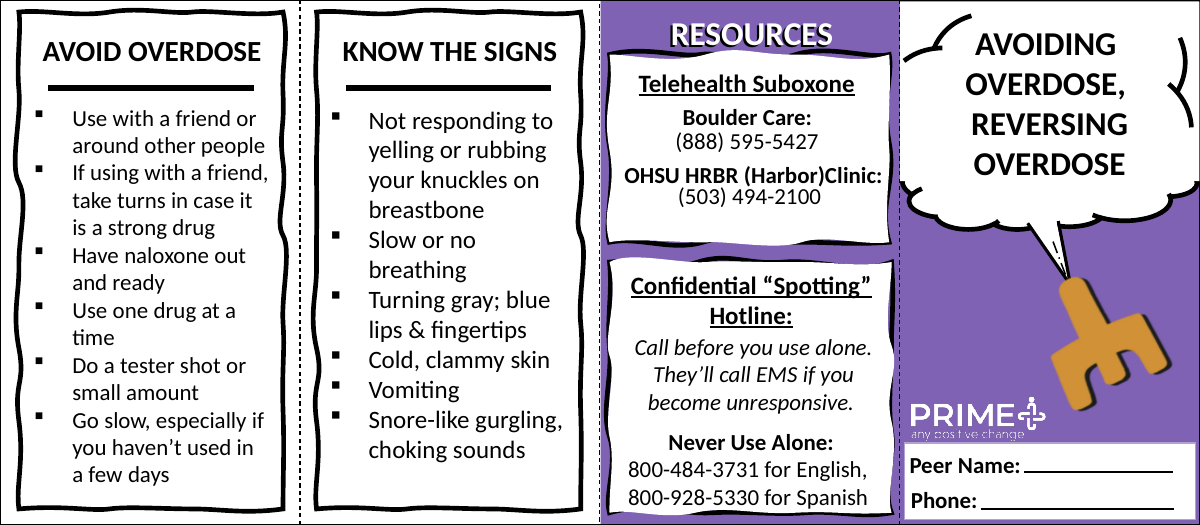

AVOIDING
OVERDOSE,
REVERSING
OVERDOSE
Peer Name:
Phone:
RESOURCES
RESOURCES
Telehealth Suboxone
Boulder Care:
(888) 595-5427
OHSU HRBR (Harbor)Clinic:
(503) 494-2100
Confidential “Spotting” Hotline:
Call before you use alone. They’ll call EMS if you become unresponsive.
Never Use Alone:
800-484-3731 for English,
800-928-5330 for Spanish
AVOID OVERDOSE
Use with a friend or around other people
If using with a friend, take turns in case it is a strong drug
Have naloxone out and ready
Use one drug at a time
Do a tester shot or small amount
Go slow, especially if you haven’t used in a few days
KNOW THE SIGNS
Not responding to yelling or rubbing your knuckles on breastbone
Slow or no breathing
Turning gray; blue lips & fingertips
Cold, clammy skin
Vomiting
Snore-like gurgling, choking sounds

## Slide 2
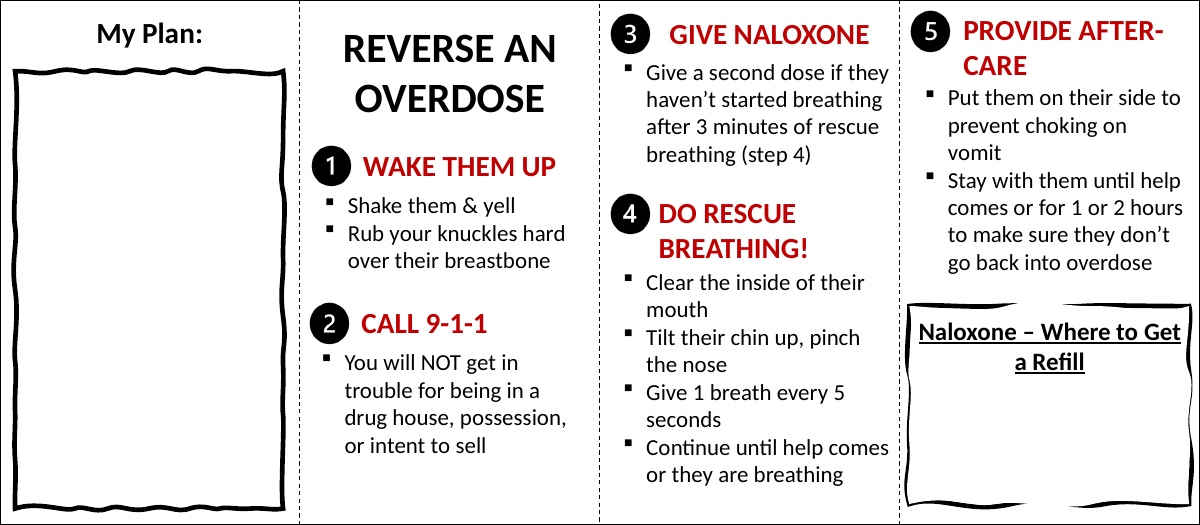

PROVIDE AFTER-CARE
Put them on their side to prevent choking on vomit
Stay with them until help comes or for 1 or 2 hours to make sure they don’t go back into overdose
GIVE NALOXONE
Give a second dose if they haven’t started breathing after 3 minutes of rescue breathing (step 4)
REVERSE AN OVERDOSE
WAKE THEM UP
Shake them & yell
Rub your knuckles hard over their breastbone
DO RESCUE BREATHING!
Clear the inside of their mouth
Tilt their chin up, pinch the nose
Give 1 breath every 5 seconds
Continue until help comes or they are breathing
CALL 9-1-1
You will NOT get in trouble for being in a drug house, possession, or intent to sell
My Plan:
Naloxone – Where to Get a Refill

## Slide 3
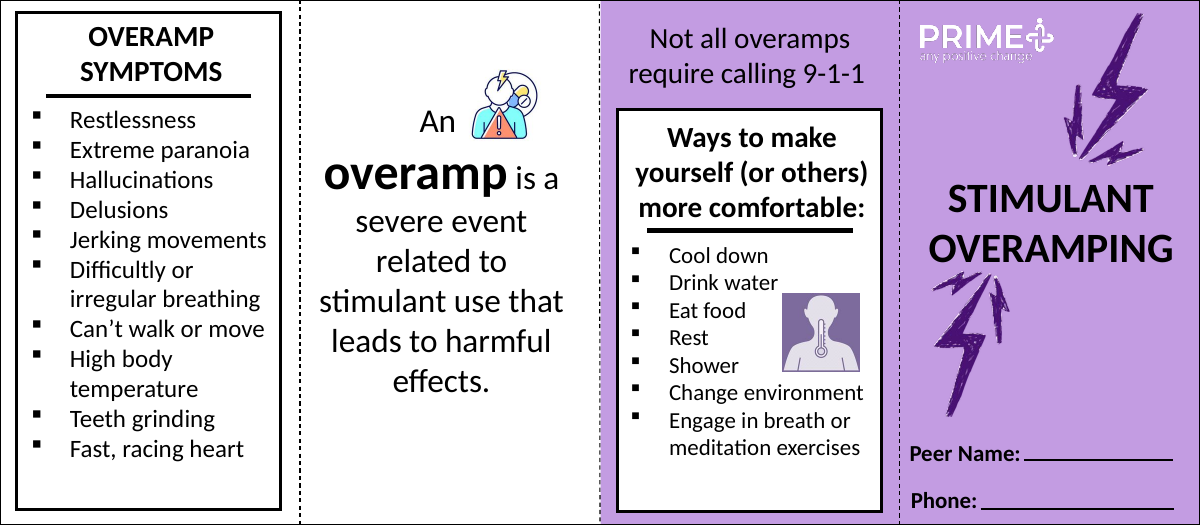

STIMULANT OVERAMPING
Peer Name:
Phone:
OVERAMP SYMPTOMS
Restlessness
Extreme paranoia
Hallucinations
Delusions
Jerking movements
Difficultly or irregular breathing
Can’t walk or move
High body temperature
Teeth grinding
Fast, racing heart
Not all overamps require calling 9-1-1
Ways to make yourself (or others) more comfortable:
Cool down
Drink water
Eat food
Rest
Shower
Change environment
Engage in breath or meditation exercises
An
overamp is a severe event related to stimulant use that leads to harmful effects.

## Slide 4
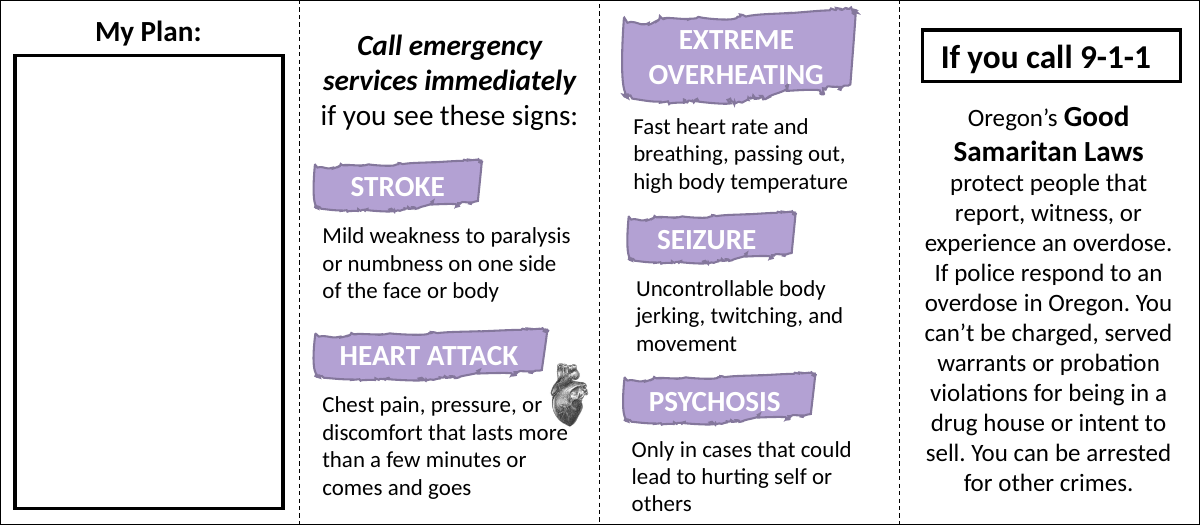

My Plan:
EXTREME OVERHEATING
Fast heart rate and breathing, passing out, high body temperature
Call emergency services immediately if you see these signs:
STROKE
Mild weakness to paralysis or numbness on one side of the face or body
SEIZURE
Uncontrollable body jerking, twitching, and movement
HEART ATTACK
Chest pain, pressure, or discomfort that lasts more than a few minutes or comes and goes
PSYCHOSIS
Only in cases that could lead to hurting self or others
If you call 9-1-1
Oregon’s Good Samaritan Laws protect people that report, witness, or experience an overdose. If police respond to an overdose in Oregon. You can’t be charged, served warrants or probation violations for being in a drug house or intent to sell. You can be arrested for other crimes.
