# Supplementary material for: Centering peers in design and training for a peer-delivered contingency management program for self-identified harm reduction and treatment goals
Source: Harm Reduct J. 2025 May 6;22(Suppl 1):72. doi: 10.1186/s12954-025-01213-z (PMC12057027; doi:10.1186/s12954-025-01213-z)

| **Overview information to share with all participants** [Can use like a script] | **More details for peer and/or to share with participant as appropriate** *[For example, if participant has time, remains engaged, or has follow-up questions]* |
| --- | --- |
| **Introduction** | |
| Thanks for meeting to work on this. Like I said, this can take a while, but we can take a break whenever you need to. First, we’ll talk about important things to know about overdoses, then we’ll work on your overdose prevention plan, then we’ll do a Narcan/naloxone training refresher. If you have questions or anything is unclear, feel free to stop me. It will take us about XX minutes to get through all this information. | *[****Peer ACTION****: Repeated discussions with participants about their opioid overdose plan is important.]* |
| **Naming the issue, asking questions** | |
| First, I’d like to **better understand what you already know** about fentanyl and other drugs involved in overdoses in Oregon.   - What have you heard or seen about the increase in overdoses in Oregon? - Do you have any concerns about overdosing?   I am interested to hear more about any experiences you have had **witnessing an overdose**.   - What’s your experience with being around someone who was overdosing? - Do you have an idea why it happened? - Is there anything you wish you would have known or have done differently in the moment?   Thank you for sharing. I also want to hear about any **experiences you have had with overdosing**.   - You mentioned you’ve overdosed before. What was your experience? - [About the last overdose:] What do you think happened that led to that overdose? - Is there anything you would like to or have changed to not get to that spot again? | Overdoses have gone up a lot, especially now that fentanyl is here. People are most often dying from methamphetamine, fentanyl, or a mix of the two. Stimulants can also be contaminated with fentanyl without the person knowing it. Other drugs like benzos are also mixed in with overdoses more often now. |
| **Sharing information** | |
| Let’s talk about **some** **things that may increase the chance of overdosing**. Some things that increase overdose risk are:   - Using more or stronger opioids - Having overdosed before - Mixing opioids with other drugs like benzos, alcohol, or stimulants - Using alone (including in a bathroom by yourself, even if there are others inside the building) so no one is there to help you if you do overdose - Using different opioids than usual or from a different connection - Not doing a tester - Relying on others to prepare your drugs and/or to inject you - Using the same amount the day you leave detox, treatment, jail, or the hospital as the day you went into detox, treatment, jail or the hospital. (It is really important to do a tester after any period of days that you haven’t used opioids). | [***Peer ACTION****:* *Give participant the handout with info. The participant can read along or just listen and discuss verbally, whichever they prefer. They can also take it with them if they want.]*  Using fentanyl, even if you know that you are using it. (Fentanyl is about 4 times riskier for overdose than injecting heroin.) |
| Now let’s talk about **ways to reduce overdose risk**.  **What are some of things that you do to protect yourself from overdose?**  Thank you for sharing your current strategies to prevent overdose. Some other ways to prevent overdose include personal and buddy system planning. **Here is an overdose prevention plan wallet-card** for you to identify ways that seem like good options for you to do to keep yourself safe from overdose. As I go through this list, let me know any that you’d like to include in your plan.  **Things you can do for yourself:**   - Avoid using other drugs with opioids. - Start low, go slow: Use a small amount or tester before doing the normal dose (you can always go up, you can’t go down); Watch and wait before you or the next person uses; Space out doses (give yourself time between doses) - Use a smaller amount after a break, detox, or jail. (It is really important to do a tester after any period of days that you haven’t used opioids). - Always carry Narcan/naloxone and let others know where you keep it.   - If using with others, make sure naloxone is out where people can see it.   - Know where others keep their naloxone.   - Get new naloxone every two years because it expires. - Use fentanyl test strips on drugs before using. - Learn to prepare your own drugs. - Learn how to inject yourself. - Shift from injecting to smoking: This is helpful to prevent infection/disease but be aware that with fentanyl, smoking or ingesting can be as risky.   **Here are some ways to make sure someone can help you if you need:**   - If you can, use only while someone is right there with you   - Make sure someone has Narcan/naloxone and a phone to call for help.   - Take turns, do not all use at the same time, so someone is awake in case it’s a strong drug. - Set an alarm or time that other people can hear and will respond to if you overdose. - Have a friend or someone you trust check on you (text, call, or come by). - Use in a place where someone is likely to find you if you need help. - Use a confidential service like these:   - Never Use Alone .com: call 800-484-3731 or for Spanish: 800-928-5330: An all-volunteer peer-run phone-based “spotter” service available 24/7/365 for people who are using drugs while alone: If a person becomes non-responsive while dialed in, peer support operators call EMS on their behalf to come to the person’s location.   - The Brave app: [www.brave.coop/overdose-detection-app](http://www.brave.coop/overdose-detection-app): An app you download. You list who you want to be contacted if you overdose (911, a roommate or friend, etc.), and you connect to a live supporter through the app before you use drugs alone. | |
| **Making a personal overdose prevention plan** | |
| **We already started talking about ways to reduce your risk of an overdose. Let’s make sure we have them all written down.**  I’m also going to keep a copy of your plan so I can help you remember later if you need reminders or another copy.  Let’s look back over the strategies on the handout.   - Any others to add? - Which things do you want to start doing that you haven’t done before? - Let’s make sure we write down something in both personal strategies and buddy systems. - What do you think about the Never Use Alone phone line? [If interested]: Let’s call together so you have the number in your phone. | [***Peer ACTION****:* *Review strategies already written down. Discuss any additional options that you think would be important for the participant to consider including. ]* |
| **Next, let’s chat about Narcan/naloxone.**   - Do you have naloxone? - Where do you keep it? - Are there other people in your life who you think should have naloxone? - What will you do if you need a refill? | - [If available]: Are you interested in trying out fentanyl test strips?   [***Peer ACTION****:* *It will be important to ask at later visits how the strategies on their personal plan are going. The goal of these conversations is to see how people are doing on using their chosen prevention strategies. If the strategies aren’t working, you can help the participant think through what might work better.]* |
| **Providing a naloxone rescue training** | |
| Next, let’s **do a naloxone training or refresher**.   - First, what do you know about the signs of an overdose? - What should you do if you see the signs of an overdose?   Great, you already know a lot. Let’s step through each step together to help you and other stay safe. Here are the steps if think someone is having an overdose:  **Step 1: Try to wake them up**   - Shake them and yell. - Grind your knuckles HARD back and forth into their breastbone. - Check for breathing. - Do a “Verbal Narcan”: Say, “If you can’t wake up, I’m going to Narcan you!”   **Step 2: Call 9-1-1**   - Calling for an ambulance is important because the person can keep going back into overdose and they can also have other health problems because of the overdose that need to be checked. - You can say: “My friend stopped breathing and is turning blue!”   - You don’t need to say that any drugs were used until the ambulance arrives.   Step 3: Give naloxone  Do you know how to give naloxone? I can do a demonstration.   - Remember, naloxone only lasts about 1 hour, so the effects of the drug they used may come back and they may go back into overdose. - You might need to give a second dose if they haven’t started breathing after 3 minutes of rescue breathing (step 4)   **Step 4: Do rescue breathing**   - Rescue breathing is one of the most important steps. Rescue breathing is the quickest way to get oxygen into someone and might be the difference in surviving an overdose, especially if xylazine (tranq) or fentanyl are involved.. - Check that there’s nothing in their mouth. - Tilt the person’s head back to straighten out the airway, pinch their nose. - Give 1 breath into their mouth every 5 seconds. - Continue until help comes or they start breathing. Try to stay calm and use normal breaths (don’t over-breathe) so that you can keep going for a long time if needed. - If still no breathing after 3 minutes, give another dose of naloxone. Then continue rescue breathing.   **Step 5: Provide after-care**   - Stay with the person until help comes or they are awake for at least 2 to 3 hours. If the person goes back into overdose, repeat the steps. - Encourage the person not to use again for at least 1 hour after naloxone is administered. Remind them that naloxone will wear off in a little while and they will stop feeling dopesick. - If you need to stop or step away, put the person in the recovery position. - Be gentle with them and yourself afterwards! | **Signs of an overdose:**   - Not responding to yelling or stimulation, like rubbing your knuckles up and down the breastbone - Breathing is slow, shallow, or stopped - Pulse (heartbeat) is slow, erratic or not there - For lighter skinned people, skin tone turns bluish or pale - For darker skinned people, skin tone turns grayish or ashen - Cold, clammy skin - Fingernails and lips turn blue or purple - Body is limp - Vomiting - Snore-like gurgling or choking sounds   **Good Samaritan Laws** protect people that report, witness, or experience an overdose. If police respond to an overdose in Oregon:   - You will NOT be arrested or prosecuted for the following crimes:   - Being present at a drug house   - Possession of drugs or paraphernalia with intent to sell, if the evidence was obtained as part of emergency response - You will NOT be served for pre-existing arrest warrants for these crimes - You will NOT be served for a parole/probation violation for these crimes - You CAN be arrested for other crimes or other warrants |


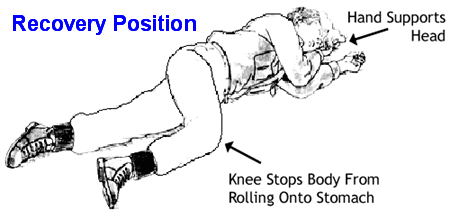

Supplement: Supplementary file 2 — Additional file 2. [file 12954_2025_1213_MOESM2_ESM.docx]
