# Supplementary material for: Centering peers in design and training for a peer-delivered contingency management program for self-identified harm reduction and treatment goals
Source: Harm Reduct J. 2025 May 6;22(Suppl 1):72. doi: 10.1186/s12954-025-01213-z (PMC12057027; doi:10.1186/s12954-025-01213-z)
Supplement: Supplementary file 3 — Additional file 3. [file 12954_2025_1213_MOESM3_ESM.docx]

| **Overview information to share with all participants** [Can use like a script] | **More details for peer and/or to share with participant as appropriate** *[For example, if participant has time, remains engaged, or has follow-up questions]* |
| --- | --- |
| **Introduction** | |
| In this training, we’re going to talk about stimulant overamping. We’ll talk about what an overamp is, go over symptoms and prevention strategies and then create a personal prevention plan. If you need to take a break at any point let me know. If you have questions or anything is unclear, feel free to stop me. It will take us about 20 minutes to get through all this information. | *[****Peer ACTION****: Repeated discussions with participants about their stimulant overamping plan is important.]* |
| **Naming the issue, asking questions** | |
| First, I’d like to **better understand what you already know** about stimulant overamping.   - What are some things you know, or think about overamping? - Is it something you have been concerned or think about? | From 2009 to 2020, there was an over 1000% increase in deaths due to overamping.  [***Peer ACTION****:* *Give participant the handout with info. The participant can read along or just listen and discuss verbally, whichever they prefer. They can also take it with them if they want.]* |
| There is **no single definition of overamping.** Broadly, overamp is a severe event related to stimulant use that leads to harmful effects.  Overamping is also called a stimulant overdose or stimulant toxicity. Overamp symptoms are different from an opioid overdose, but both can cause brain damage or death. | - A person having a stimulant overamp is usually conscious, breathing quickly, and overheating. They may lose consciousness in later stages. - A person having an opioid overdose is usually barely breathing or not breathing at all and is nonresponsive or unconscious. |
| Let’s go over some of the **symptoms of overamping**. Some symptoms are mental, and some are physical. During an overamp, you may experience one or more of these symptoms.  **Some mental symptoms include** confusion, restlessness, hypervigilance, intense panic.  **Some physical symptoms include** jerking movements, chest pain or tightness, unable to stay still, difficult or irregular breathing. I’m curious about any experiences you may have had with overamping. You may have experienced an overamp and not have known.  - Can you think of a time when you or a friend ever took too much [meth/cocaine/crack]? - What was that like, what did it feel like in your body? - What do you wish you could have changed in that situation? - What changed for you after that situation? - How do you feel about responding to overamping (either for yourself or helping someone else)? | **Mental Symptoms (longer list)**   - Confusion - Restlessness - Hypervigilance - Intense panic - Increased aggressiveness - Hallucinations/delusions - Extreme paranoia - Extreme agitation - Suicidal ideation   **Physical Symptoms (longer list)**   - Jerking movements - Chest pain or tightness - Being unable to stay still - Difficulty or irregular breathing - Painful headache - Can’t walk or move - Can’t feel arms or legs - High body temp (sweating; hot, dry skin) - Uncontrollable teeth grinding - Fast, racing heart - Seizure or shaking you can’t control - Heart attack |
| **Sharing information** | |
| Let’s talk about **some** **things that may increase the chance of overamping**. Some things that increase risk of overamping are:   - Using higher doses than you are used to - Lack of sleep, especially multiple days of little or no sleep - Using for multiple days, especially without sleeping - Using in an unfamiliar environment, or place you don’t feel comfortable in - Using in different ways: You may feel more uncomfortable when you inject vs smoke - If you are sick your body may not handle drugs like it normally does. - If you have heart problems, you may be at an increased risk of a medical emergency, like heart attack.   **Do you have any current medical conditions**, for example cardiovascular or psychological, that you think may put you at increased risk? | [***Peer ACTION****:* *If participant reports current medical condition putting them at increased risk, encourage them to get care immediately.]* |
| There are lots of **ways for you to reduce the risk of overamping**, such as:   - Getting enough sleep - Drinking water and eating meals; remember: energy drinks and coffee are not food - Taking breaks from using - Taking your prescribed medications, especially to protect your heart - Using in a place and with people you feel comfortable   That is a lot to remember! Most important: sleep, drink water, and eat food.  **Repeated overamps can be especially harmful**. Reducing or eliminating overamps can help prevent long-term impacts and severe cardiovascular outcomes, like heart attack, stroke, heart failure or irregular heartbeat, and prevent severe and chronic psychosis.  Some people may enjoy overamping after-effects, but they can lead to harm over time. Limiting binges or multiple runs can be a way to reduce harms associated with stimulant use. Try to “start low and go slow” – take a small dose and wait awhile before taking more.   - Which of these ways to prevent overamping do you think would work for you? - Which are things you already do? Which are things you want to start doing? | **More details about why risk reduction actions are important:**   - Lack of sleep increases overamp risk because even without drugs, little to no sleep can make you feel unwell. - Take a break and take care of yourself (sleep, eat, shower). - Stay hydrated and eat: drinking plenty of fluids and eating meals can help your body feel better overall. - Be with people who know you and can tell if you need help. - If you’re using somewhere new or with new people, reducing your dose and taking it slow can make it easier to check in with yourself about how you feel. - Limit combos: combining stimulants with other drugs can change the effects of each individual drug. - Pay attention to dose! If you have a scale, weighing out can help you notice a dose amount that makes you uncomfortable. - Take any medications you’re prescribed, especially if you have heart problems or high blood pressure. - Stimulants last a long time in your body. If you feel close to your limit, don’t use more. |
| Even though we are talking about stimulants, it’s important to also **think about co-use of stimulants and opioids**. There's been an increase in overdose deaths from stimulants and fentanyl mixed together.  People wo use stimulant **should carry Narcan/naloxone**. | - Sometimes people mix drugs on purpose, or sometimes stimulants can be contaminated with fentanyl without the person knowing it. - [If the person didn't do the opioid overdose planning] We can do a Narcan training together if you'd like. |
| **Making a personal overamping prevention plan** | |
| **We already started talking about ways to reduce your risk of an overamp. Let’s go ahead and write down things you want to do to reduce your risk.** We have these half-page plan templates that fold up wallet-size. | *[Note to peer: Adequate sleep, food and water especially important for stimulant users, and may be a good starting point for personal prevention plans. Below highlights strategies that may be especially useful for certain people.]*   - **Newer users:** avoid/limit binge runs, use one stimulant at a time, don’t use alone - **Long-term users** (and those who have experienced an overamp): Take care of overall health, use smaller doses, take breaks - **People with history of cardiovascular or psychiatry conditions**: Connect to a health care provider, take prescribed medication as directed, have regular check-up / steps to monitor health condition |
| **Let’s write down some things to keep you and others safe if you do experience an overamp.**  First thing to ask yourself: **Is this an emergency or not?** For example, are the symptoms life-threatening or is it enough to take steps to get more comfortable?  **Call an ambulance if you see these signs:**   - **Stroke**: extremely slurred words; droopy or numb face; sudden numbness on one side of body; can’t move face on one side - **Heart attack**: difficult breathing and cold sweat; squeezing, pressure, or pain in the chest; lightheadedness/dizziness; pain, pressure, and tightness in the neck, shoulders, jaw, or back - **Extreme Overheating:** body temp over 104 degrees; pass out or confused; fast heart rate and breathing - **Seizures:** uncontrollable body jerking, twitching, & movement; drooling/frothing at the mouth; sudden falling/loss of consciousness; loss of bladder control; breathing slows down or stops - **Psychosis that could lead to hurting self or others**: Immediate danger, like using a knife.   **If someone has passed out and is not breathing, do CPR if you are trained.**  Not all overamps require calling 911. **Here are steps to reduce discomfort** if there is not a life-threatening emergency:   - **Cool down:** ice packs or a cool towel under armpits and knees; use a fan - **Drink water** or sports drink (Gatorade)   - Avoid drinks with caffeine - **Eat food:** easier to eat the better; high salt - **Rest:** Take a nap, close your eyes, or simply lay/sit down somewhere comfy - **Shower:** A cool or warm shower can help bring some physical and mental relief - **Change environment:** move to a more comfortable place or take a walk - **Engage in breath or meditation exercises** - **Physical contact:** massage self or ask someone else   **Ways to help others who may be overamping**   - Be calm and help the person be calm - Try to get them to slow down and rest - Give them water and keep them hydrated - Try to help them cool down   **After experiencing an overamp,** you may feel tired, confused, and sad. Take time to rest and check-in with yourself and others. Some options:   - Take a shower - Eat a good meal - Stay hydrated: water and Gatorade (continue to avoid caffeine) - Take supplements for calming (L- theanine and magnesium glycinate or gluconate) - Take herbal tinctures/supplements for help falling asleep: skullcap, valerian, kava, chamomile, passionflower (supplements can interact with some medications so read labels and talk with a doctor when possible) | - **Strokes** happen very quickly; when a blood clot blocks the blood from moving in the body and when bleeding in the brain occurs. - **Heart attacks** look very similar no matter the cause. It can be hard to tell if a heart attack is happening. If you see some signs happen at the same time or your friend has a heart condition, call an 9-1-1 immediately. - **Heart attack symptoms for women:** The most common heart attack symptoms are the same in men and women — chest pain, pressure or discomfort that lasts more than a few minutes or comes and goes. However, women often describe heart attack pain as pressure or tightness. It's possible to have a heart attack without chest pain. Women are more likely than men to have heart attack symptoms unrelated to chest pain, such as:   - Neck, jaw, shoulder, upper back, or upper belly (abdomen) discomfort   - Shortness of breath   - Pain in one or both arms   - Nausea or vomiting   - Sweating   - Lightheadedness or dizziness   - Unusual fatigue   - Heartburn (indigestion) - **Extreme Overheating:** Just like a high fever, an extremely high body temperature for long periods of time can cause organ failure and brain damage. - **Seizures:** If a seizure happens, stay calm and make sure you move anything that can hurt someone out of the way (chairs, tables, etc.). Keep an eye on them in case they start choking or vomiting. DO NOT try to restrain the person. DO NOT force anything into their mouth. Seizures are an emergency if it’s the first time it’s ever happened OR multiple keep happening back-to-back. - **Psychosis that could lead to hurting yourself or someone else:** Psychosis where someone is trying to hurt themselves or someone else is extremely rare. Most people will not need medical care or be dangerous. Psychosis will often decrease after a few hours. It may take longer for some people.   **Good Samaritan Laws** protect people that report, witness, or experience an overdose. If police respond to an overdose in Oregon:   - You will NOT be arrested or prosecuted for the following crimes:   - Being present at a drug house   - Possession of drugs or paraphernalia with intent to sell, if the evidence was obtained as part of emergency response - You will NOT be served for pre-existing arrest warrants for these crimes - You will NOT be served for a parole/probation violation for these crimes - You CAN be arrested for other crimes or other warrants   [***Peer ACTION****:* *It will be important to ask at later visits how the strategies on their personal plan are going. The goal of these conversations is to see how people are doing on using their chosen prevention strategies. If the strategies aren’t working, you can help the participant think through what might work better.]* |
