# Supplementary material for: Centering peers in design and training for a peer-delivered contingency management program for self-identified harm reduction and treatment goals
Source: Harm Reduct J. 2025 May 6;22(Suppl 1):72. doi: 10.1186/s12954-025-01213-z (PMC12057027; doi:10.1186/s12954-025-01213-z)
Supplement: Supplementary file 4 — Additional file 4. [file 12954_2025_1213_MOESM4_ESM.docx]

## What is an overdose?

An opioid overdose happens when a toxic amount of opioids or a combination of opioids and other drugs overwhelm the body.

## What are the signs of an overdose?

A person may be experiencing an overdose if they are:

- Unresponsive to yelling or stimulation, like rubbing your knuckles up and down the sternum
- Breathing slow, shallow, or not at all
- Having a slow, erratic, or no pulse
- Turning pale, blue or gray (lips and fingernails)
- Having cold, clammy skin
- Limp
- Vomiting
- Making snoring, gurgling, or choking sounds

## What increases the risk of overdosing?

- Using more or stronger opioids
- Having overdosed before
- Mixing opioids with other drugs like benzos, alcohol, or stimulants
- Using alone (including in a bathroom by yourself, even if there are others inside the building) so no one is there to help you if you do overdose
- Using different opioids than usual or from a different connection
- Not doing a tester
- Relying on others to prepare your drugs or to inject you
- Using the same amount the day you leave detox, treatment, jail, or the hospital as the day you went into detox, treatment, jail or the hospital.

## How can you reduce the risk of overdosing?

You can reduce your risk of overdose by using some personal strategies and setting up a buddy system to make sure someone can help you if you need it.

| **Personal strategies** | **Buddy system** |
| --- | --- |
| - Avoid using other drugs with opioids - Start low, go slow:   - Use a small amount or tester before doing the normal dose   - Watch and wait before you or the next person uses   - Take a break between doses - Use a smaller amount after a break, detox, or jail - Always carry naloxone and let others know where you keep it   - When using, make sure naloxone is out where people can see it   - Know where others keep their naloxone   - Get new naloxone every two years because it expires - Use fentanyl test strips on drugs before using - Learn to prepare your own drugs - Learn how to inject yourself - Shift from injecting to smoking, which can be helpful to prevent infection and disease but be aware that with fentanyl, smoking or ingesting can be as risky | - If you can, use only while someone is right there with you - Make sure someone has naloxone and a phone to call for help - Take turns using - Set an alarm or time that other people can hear and will respond to if you overdose. - Have a friend or someone you trust check on you (text, call, or come by) - Use in a place where someone is likely to find you if you need help - Use a confidential service like Never Use Alone or the Brave App:   **Never Use Alone:** NeverUseAlone.com or call 800-484-3731 or for Spanish: 800-928-5330   - An all-volunteer peer-run phone-based “spotter” service available 24/7/365 for people who are using drugs while alone - If a person becomes non-responsive while dialed in, peer support operators call EMS to come to the person’s location   **The Brave App:** www.brave.coop/overdose-detection-app   - An app you download. You list who you want to be contacted if you overdose (911, a roommate or friend, etc.), and you connect to a live supporter through the app before you use drugs alone. |
