# Supplementary material for: Centering peers in design and training for a peer-delivered contingency management program for self-identified harm reduction and treatment goals
Source: Harm Reduct J. 2025 May 6;22(Suppl 1):72. doi: 10.1186/s12954-025-01213-z (PMC12057027; doi:10.1186/s12954-025-01213-z)
Supplement: Supplementary file 5 — Additional file 5. [file 12954_2025_1213_MOESM5_ESM.docx]

## What is an overamp?

There is no single definition of overamping. Broadly, overamp is a **severe event related to stimulant use** that leads to harmful effects.

Overamping is also called a stimulant overdose or stimulant toxicity. Overamp symptoms are different from an opioid overdose, but both can cause brain damage or death.

## **What are the symptoms of an overamp?**

Some symptoms are mental, and some are physical. During an overamp, you may experience one or more of these symptoms.

| **Mental symptoms** | **Physical symptoms** |
| --- | --- |
| - Confusion - Restlessness - Hypervigilance - Intense Panic - Increased Aggressiveness - Hallucinations/delusions - Extreme Paranoia - Extreme Agitation - Suicidal Ideation | - Jerking movements - Chest pain or tightness - Difficulty or irregular breathing - Really painful headache - Can’t walk or move - Can’t feel arms or legs - High body temperature - Teeth grinding - Fast, racing heart - Seizure or shaking you can’t control |

## What increases the risk of overamping?

- Using higher doses than you are used to
- Lack of sleep, especially multiple days of little or no sleep
- Using for multiple days, especially without sleeping
- Using in an unfamiliar environment, or place you don’t feel comfortable in
- Using in different ways: You may feel more uncomfortable when you inject vs smoke
- If you are sick your body may not handle drugs like it normally does.
- If you have heart problems, you may be at an increased risk of a medical emergency, like heart attack.

## How can you reduce the risk of overamping?

- Getting enough sleep
- Drinking water and eating meals; remember: energy drinks and coffee are not food
- Taking breaks from using
- Taking your prescribed medications, especially to protect your heart
- Using in a place and with people you feel comfortable

## When should you call 9-1-1?

Call an ambulance if you see these signs:

- **Stroke**: extremely slurred words; droopy or numb face; sudden numbness on one side of body; can’t move face on one side
- **Heart attack**: difficult breathing and cold sweat; squeezing, pressure, or pain in the chest; lightheadedness/dizziness; pain, pressure, and tightness in the neck, shoulders, jaw, or back
- **Extreme Overheating:** body temp over 104 degrees; pass out or confused; fast heart rate and breathing
- **Seizures:** uncontrollable body jerking, twitching, and movement; drooling/frothing at the mouth; sudden loss of consciousness; loss of bladder control; breath slows down or stops
- **Psychosis that could lead to hurting self or others**: Immediate danger, like using a knife.

## What should you do if there is not a life-threatening emergency?

Not all overamps require calling 911. **Here are steps to reduce discomfort** if there is not a life-threatening emergency:

- **Cool down:** ice packs or a cool towel under armpits and knees; use a fan
- **Drink water** or sports drink (Gatorade); Avoid drinks with caffeine
- **Eat food:** easier to eat the better; high salt
- **Rest:** Take a nap, close your eyes, or simply lay/sit down somewhere comfy
- **Shower:** A cool or warm shower can help bring some physical and mental relief
- **Change environment:** move to a more comfortable place or take a walk
- **Engage in breath or meditation exercises**
- **Physical contact:** massage self or ask someone else

## What should you do after you or someone else experiences an overamp?

**After experiencing an overamp,** you may feel tired, confused, and sad. Take time to rest and check-in with yourself and others. Some options:

- Take a shower
- Eat a good meal
- Stay hydrated: water and Gatorade (continue to avoid caffeine)
- Take supplements for calming (L- theanine and magnesium glycinate or gluconate) and for help falling asleep: skullcap, valerian, kava, chamomile, passionflower (supplements can interact with some medications so read labels and talk with a doctor when possible)
